# Measurement of operative femoral anteversion during cementless total hip arthroplasty and influencing factors for using neck-adjustable femoral stem

**DOI:** 10.1186/s13018-021-02506-2

**Published:** 2021-05-31

**Authors:** Jingyang Sun, Bohan Zhang, Lei Geng, Qingyuan Zheng, Juncheng Li, Wenzhe Cao, Ming Ni, Guoqiang Zhang

**Affiliations:** 1 Medical School of Chinese PLA, Beijing, 100853 China; 2 Department of Orthopedics, the First Medical Center, Chinese People’s Liberation Army General Hospital, Fuxing Road, Haidian District, Beijing, 100853 China

**Keywords:** Femoral anteversion, Total hip arthroplasty, Operative, Measurement

## Abstract

**Background:**

Placement of femoral stem in excessive anteversion or retroversion can cause reduced range of motion, prosthetic impingement, and dislocation. The aim of this study was to assess the operative femoral anteversion in patients treated with total hip arthroplasty (THA) and analyze the need of adjusting stem anteversion.

**Methods:**

We retrospectively included 101 patients (126 hips) who underwent cementless THA with a manual goniometer to determine the femoral anteversion between October 2017 and December 2018. The operative femoral anteversion we measured was recorded during THA. We further divided those hips into three subgroups based on the range of operative femoral anteversion: group 1 (<10°), group 2 (10–30°), and group 3 (>30°) and compared the differences of their demographic data. Univariate and multivariate logistic regression were used to identify the influencing factors for the need of neck-adjustable femoral stem. The clinical and radiographic outcomes were also assessed. Perioperative complications were recorded.

**Results:**

After THA, the Harris hip scores improved from 52.87 ± 15.30 preoperatively to 90.04 ± 3.31 at the last follow-up (*p* < 0.001). No implant loosening, stem subsidence, and radiolucent lines were observed on radiographs. No severe complications occurred and no components needed revision at the latest follow-up. The mean operative femoral anteversion was 14.21° ± 11.80° (range, −9 to 60°). Patients with femoral anteversion more than 30° were about 10 years younger than others. Femoral anteversion >30° was more common in patients with developmental dysplasia of the hip (DDH). There were totally 14 hips treated with the neck-adjustable femoral stem. From the univariate analysis, we can observe that female sex, diagnosis of DDH (compared with osteonecrosis), and higher operative femoral anteversion and its value >30° (compared with <10°) are associated with higher rates of using the neck-adjustable femoral stem. However, all these factors were no longer considered as independent influencing factors when mixed with other factors.

**Conclusions:**

This study highlighted the significance of operative femoral anteversion. Identification of abnormal femoral anteversion could assist in adjusting stem anteversion and reduce the risk of dislocation after THA.

## Background

Proper positioning of both acetabular and femoral components can lower the rate of impingement, dislocation, and accelerated wear in patients with total hip arthroplasty (THA) [1–3]. Many studies focused on the orientation of the acetabular component in THA, but little has been conducted regarding the position of the femoral component [3–5]. Even though the stem version is not as important as the cup version after THA, placement of femoral stem in excessive anteversion or retroversion can cause a clinically relevant reduction in range of motion and increase in the incidence of dislocation [6]. Since more postoperative dislocation occurred within Lewinnek’s safe zone, the concept of combined anteversion gained its popularity [5, 7, 8]. No matter femur first or cup first technique, it is necessary to know the native anteversion of the femur before implanting the femoral stem.

Several methods have been introduced to determine the femoral anteversion. The most commonly used clinical method to assess femoral anteversion is Craig’s test, which is based on differences between medial and lateral rotation on the extended hip [9]. The means of radiographic evaluation contains X-ray under special position and CT and MRI scan [10–12]. The latter two which involve the profile of the distal femur are thought to be more accurate and comprehensive. However, the preoperative evaluation pays more attention to the femoral neck anteversion, which is not equal to the torsion of the intramedullary canal [13, 14]. Therefore, even knowledge of the neck anteversion based on CT scan, it would be better to measure the version on the cutting surface of the femoral neck during operation.

The reference axis of the distal femur cannot be directly visualized during THA, so surgeons usually orient the lower leg perpendicular to the floor as a surrogate for the posterior condylar axis in order to measure the femoral anteversion [15, 16]. Unlike the definitions of anatomical, radiographic, and operative acetabular anteversion by Murray et al., there has been no specific classification of femoral anteversion [17]. When measuring the femoral anteversion with this method, we prefer to call it “operative anteversion” to distinguish from femoral neck anteversion. However, several studies demonstrated that visual estimation of the femoral anteversion had poor precision even for experienced surgeons [18, 19]. Thereafter, manual goniometers of different kinds with the same measuring principle have been reported, with an acceptable absolute error [15, 16, 20]. But these goniometers also relied on the premise that the lower leg axis was vertical to the reference axis of the distal femur, and recent studies showed that knee osteoarthritis could increase the error in estimating femoral anteversion [16, 21]. It must be acknowledged that navigation can assess femoral anteversion with high accuracy [7, 22]. However, the navigation is not available to most orthopedic surgeons and has the disadvantages of prolonged operative time and higher costs.

We designed a goniometer to measure the operative anteversion of the femur on the cutting surface during THA. We used it to identify the abnormal femoral anteversion and further to guide the implantation of the femoral stem. Therefore, the aim of this study was to assess the operative femoral anteversion in patients treated with THA and analyze the need of adjusting stem anteversion.

## Patients and methods

### Patients

We retrospectively reviewed 101 patients (126 hips, 76 hips of unilateral cases and 50 hips of bilateral cases) who underwent cementless THA with a goniometer to determine the femoral anteversion between October 2017 and December 2018. Inclusion criteria were primary THA, severely symptomatic hip, and lowered life quality. Patients with severe angulation deformity of the femur, active infection, or advanced knee osteoarthritis were excluded. Within this cohort, the diagnosis was osteoarthritis secondary to developmental dysplasia of the hip (DDH) in 39, osteonecrosis of the femoral head in 49, ankylosing spondylitis in 19, rheumatoid arthritis in 6, slipped epiphysis of the femoral head in 6, posttraumatic arthritis in 1, sequelae of hip pyogenic arthritis during childhood in 1, and displaced femoral neck fracture in 5. According to Crowe classification for DDH, 19 hips were type I, 11 hips were type II, 5 hips were type III, and 4 hips were type IV. There were 66 males and 60 females. The mean age was 50 ± 14 years (range, 23–85 years). The mean body mass index (BMI) was 24.35 ± 3.78 kg/m^2^ (range, 15.55–35.49 kg/m^2^). There were 71 left hips and 55 right hips. Informed consent was obtained by all patients. The study was approved by the institutional review board and conducted according to the Declaration of Helsinki principles.

### Surgical procedure

All surgeries were performed by two senior arthroplasty surgeons under general anesthesia through a posterolateral approach. Standardized preoperative planning of the prosthesis size and position was performed based on the plain radiographs. We adopted the “cup first” technique in all hips. Aiming for secure press-fit fixation, the acetabular cup was implanted with as much host bone coverage as possible. The target orientation of the cup was 40° ± 5° inclination and 25° ± 5° anteversion.

**Fig. 1:**
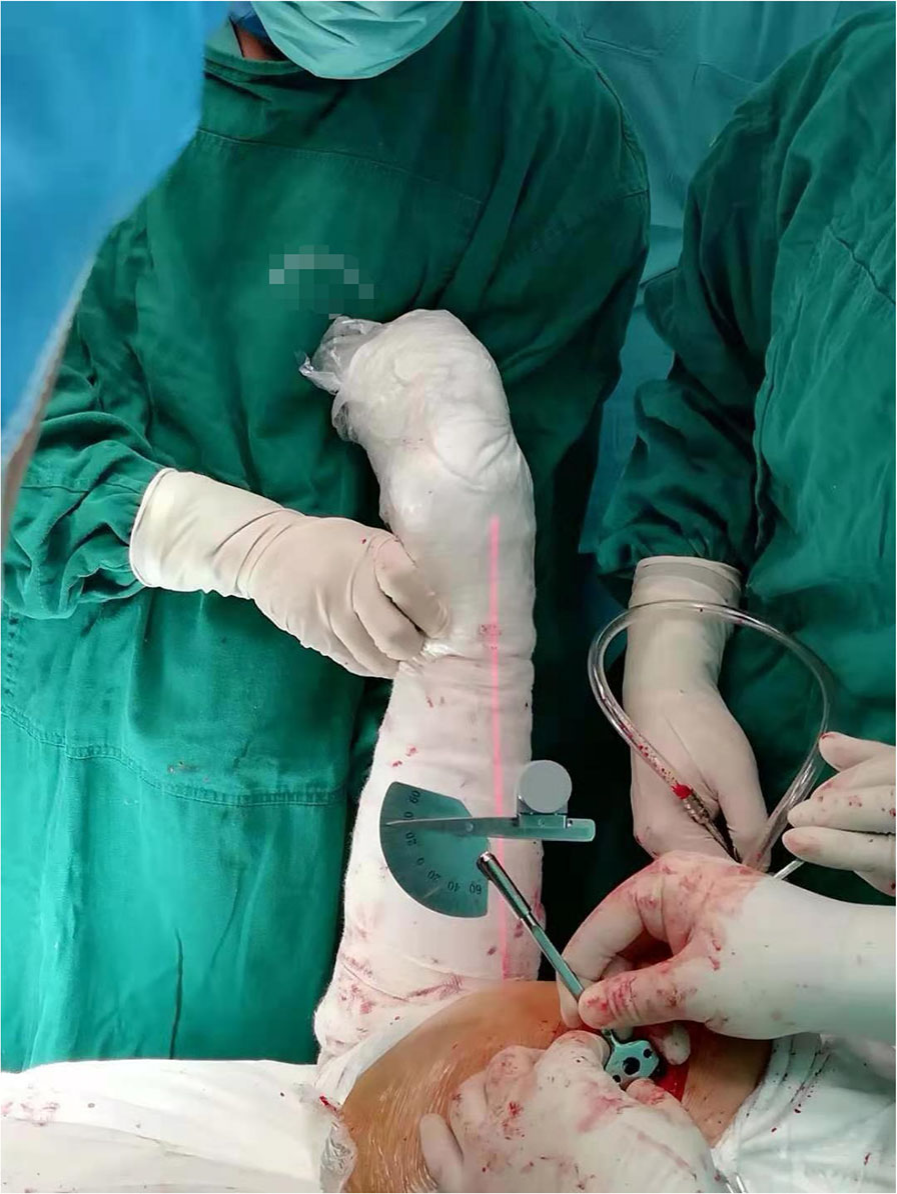
Photograph showing intraoperative measurement of the femoral anteversion using a goniometer. The goniometer consisted of 3 parts: one end of the handle was placed along the long axis of the cutting surface; the other end was a calibrated scale providing information for the orientation; and a laser device was attached to the pointer, which irradiated a ray of light to position the lower leg axis

When preparing the femoral side, the surgeon measured the anteversion of the cutting surface with a manual goniometer after the femoral neck osteotomy. The goniometer consisted of three parts: one end of the handle was placed along the long axis of the cutting surface; the other end was a calibrated scale providing information for the orientation; and a laser device was attached to the pointer, which irradiated a ray of light to position the lower leg axis (Fig. 1). The scrub technician flexed the knee and hip and internally rotated the hip until the lower leg was vertical to the operative table. In this position, the angle between the axis of the lower leg and the long axis of the cutting surface (midcortical line) was measured using the goniometer and its coangle (subtracted by 90°) was pointed on the scale indicating the femoral anteversion, which we called operative anteversion. When the femoral anteversion indicated a superior position in reference to the table, it was defined as a positive value.

Based on the target combined anteversion of 30–50° and approximate operative anteversion of cup, we assessed the difference between the measured femoral anteversion and the anticipated stem anteversion. In our opinion, a difference less than 10° can be addressed by orienting the broach version with a box chisel or rasp. Difference more than 10° increased the possibility of using a femoral stem with an optional neck version or even a modular stem. After inserting the femoral trial, the combined anteversion was measured by a coplanar test, and the soft tissue tension was also assessed. Finally, we would ensure that the stem and cup were fine-tuned with an impingement-free range of motion. The prostheses used in this cohort were shown in Table 1.

**Table 1:** Acetabular and femoral prostheses

Prostheses	Cases	Manufactures
Acetabular cup		
Betacup	60	Waldemar Link, Hamburg, Germany
Combicup	20	Waldemar Link, Hamburg, Germany
Pinnacle	12	DePuy Synthes, Warsaw, IN, USA
Trident	21	Stryker, Mahwah, NJ
Tritanium	12	Stryker, Mahwah, NJ
CDH cup	1	LDK, Beijing, China
Femoral stem		
LCU	69	Waldemar Link, Hamburg, Germany
Corail	4	DePuy Synthes, Warsaw, IN, USA
S-ROM	7	DePuy Synthes, Warsaw, IN, USA
Accolade II	39	Stryker, Mahwah, NJ
CDH stem with optional neck version	7	LDK, Beijing, China

Patients were allowed to walk with crutches on the first postoperative day. All patients received postoperative intravenous antibiotic prophylaxis with third-generation cephalosporins. Low molecular weight heparin was also administered as antithrombotic prophylaxis.

### Clinical and radiographic assessment

Patients were asked for a follow-up visit in regular intervals at 3 months, 6 months, and yearly after surgery. Clinical and radiographic assessments were performed at each visit. Clinical outcome was evaluated using the Harris hip score. Standardized digital, calibrated anteroposterior and lateral hip radiographs were acquired. The presence of implant loosening, stem subsidence, and radiolucent lines was detected by two reviewers. All perioperative complications were recorded including dislocation, implant loosening, and infection. Failure was defined as revision for any reason.

### Statistical analysis

Categorical variables were presented as frequencies and continuous variables as means and standard deviation. Paired t-test was used to evaluate differences between pre- and postoperative quantitative data. Categorical variables were analyzed using the chi-square test or Fisher’s exact test. One-way ANOVA was carried out to explore the differences between the variables in the subgroups. Binary logistic regression was used to identify the influencing factors for using the neck-adjustable femoral stem. Odds ratios (ORs) and 95% confidence intervals (CIs) were calculated for these results. All statistical analyses were performed using SPSS version 26.0 (IBM Inc., Armonk, NY). *P* values <0.05 were considered statistically significant.

### Results

The mean duration of follow-up was 30.8 ± 4.2 months (range, 24–38 months). After THA, the Harris hip scores improved from 52.87 ± 15.30 preoperatively to 90.04 ± 3.31 at the last follow-up (*p* < 0.001). No implant loosening, stem subsidence, and radiolucent lines were observed on radiographs. No severe complications occurred and no components needed revision at the latest follow-up.

**Fig. 2:**
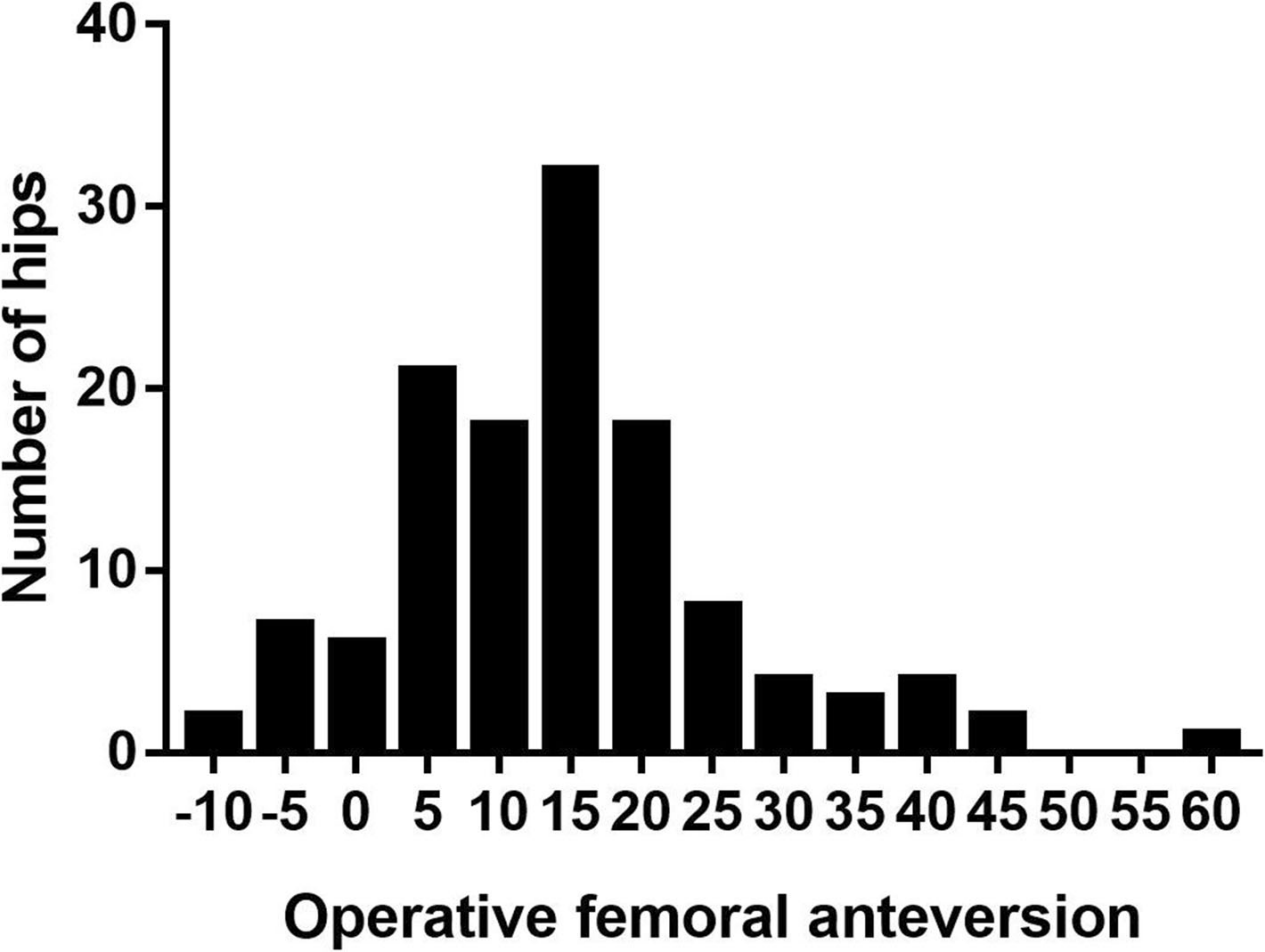
Graph of the distribution of operative femoral anteversion measured with a manual goniometer during total hip arthroplasty

The mean operative femoral anteversion was 14.21° ± 11.80° (range, −9 to 60°). A graph depicting the distribution of operative femoral anteversion is shown in Fig. 2. We further divided those hips into three subgroups based on the range of operative femoral anteversion: group 1 (<10°, 42 hips), group 2 (10–30°, 74 hips), and group 3 (>30°, 10 hips). The differences of the demographic data in these three subgroups were compared and results are shown in Table 2. It can be seen that patients with femoral anteversion more than 30° were about 10 years younger than others. With regard to different diagnoses, femoral anteversion > 30° was more common in DDH patients. Half hips in group 3 were treated with the neck-adjustable femoral stem, which was obviously greater than the other two groups.

**Table 2:** Characteristics of hips with different ranges of operative femoral anteversion

	Group 1 (<10°, 42 hips)	Group 2 (10–30°, 74 hips)	Group 3 (>30°, 10 hips)	*P*-value
Age (years)	53 ± 12	49 ± 15	40 ± 11	0.022^†,£^
Height (m)	1.66 ± 0.09	1.66 ± 0.08	1.63 ± 0.09	0.529
Weight (kg)	66.8 ± 12.7	67.9 ± 14.3	64.4 ± 8.5	0.714
BMI (kg/m^2^)	24.09 ± 3.49	24.50 ± 4.08	24.26 ± 2.80	0.857
Female/male sex, n	16/26	37/37	7/3	0.157
Diagnosis, n (%)				0.032^†,£^
DDH	10 (23.8)	22 (29.7)	7 (70)	
Osteonecrosis	15 (35.7)	34 (45.9)	0 (0)	
Ankylosing spondylitis	9 (21.4)	10 (13.5)	0 (0)	
Rheumatoid arthritis	2 (4.8)	3 (4.1)	1 (10)	
Slipped epiphysis of the femoral head	1 (2.4)	3 (4.1)	2 (20)	
Posttraumatic arthritis	1 (2.4)	0 (0)	0 (0)	
Sequelae of hip pyogenic arthritis	1 (2.4)	0 (0)	0 (0)	
Displaced femoral neck fracture	3 (7.1)	2 (2.7)	0 (0)	
Use of the neck-adjustable femoral stem	2 (4.8)	7 (9.5)	5 (50)	<0.001^†,£^

*BMI* body mass index, *DDH* developmental dysplasia of the hip

^*^p < 0.05 group 1 vs group 2; ^†^*p* < 0.05 group 1 vs group 3; ^£^*p* < 0.01 group 2 vs group 3

There were totally 14 hips treated with the neck-adjustable femoral stem (including 7 S-ROM and 7 CDH stem with optional neck version). The results of analyzing the influencing factors for the need of neck-adjustable femoral stem are shown in Table 3. From the univariate analysis, we can observe that female sex, diagnosis of DDH (compared with osteonecrosis), and higher operative femoral anteversion and its value >30° (compared with <10°) are associated with higher rates of using the neck-adjustable femoral stem. However, in the multivariate analysis, all these factors were no longer considered as independent influencing factors when mixed with other factors.

**Table 3:** Univariate analysis of the characteristics of hips treated with conventional and neck-adjustable femoral stem

	Conventional stem (112 hips)	Neck-adjustable stem (14 hips)	OR (95% CI)	*P*-value
Age (years)	50 ± 13	48 ± 18	0.991 (0.951–1.031)	0.644
Height (m)	1.66 ± 0.08	1.63 ± 0.07	0.963 (0.897–1.033)	0.292
Weight (kg)	67.7 ± 13.4	63.9 ± 12.8	0.976 (0.931–1.024)	0.322
BMI (kg/m^2^)	24.42 ± 3.76	23.78 ± 4.06	0.955 (0.819–1.113)	0.552
Female/male sex, n	48/64	12/2	8.000 (1.710–37.430)	0.008
Diagnosis, n (%)				0.026
DDH	30 (26.8)	9 (64.3)	Reference	
Osteonecrosis	48 (42.9)	1 (7.1)	0.069 (0.008–0.576)	0.013
Ankylosing spondylitis	19 (17.0)	0 (0)	0	0.998
Rheumatoid arthritis	5 (4.5)	1 (7.1)	0.667 (0.069–6.470)	0.727
Slipped epiphysis of the femoral head	4 (3.6)	2 (14.3)	1.667 (0.261–10.638)	0.589
Posttraumatic arthritis	1 (0.8)	0 (0)	0	1.000
Sequelae of hip pyogenic arthritis	1 (0.8)	0 (0)	0	1.000
Displaced femoral neck fracture	4 (3.6)	1 (7.1)	0.833 (0.082–8.433)	0.877
Operative femoral anteversion (degree)	13.06 ± 11.11	23.43 ± 13.50	1.069 (1.021–1.118)	0.004
Distribution of anteversion, n (%)				<0.001
<10°	40 (35.7)	2 (14.3)	Reference	
10–30°	67 (59.8)	7 (50)	2.090 (0.414–10.554)	0.372
>30°	5 (4.5)	5 (35.7)	20.000 (3.036–131.731)	0.002

*CI* confidence interval, *OR* odds ratio, *BMI* body mass index, *DDH* developmental dysplasia of the hip

## Discussion

Correct component placement has been considered a prerequisite for successful THA, as implant malposition directly influences postoperative stability, wear, and aseptic loosening [1–3]. Even though the stem version is not as important as the cup version after THA, it can also influence the range of motion, bone loading, and gait [6, 23, 24]. Early identification of the abnormal femoral version can assist in obtaining optimal stem anteversion in THA. In this study, we used a manual goniometer to determine the operative femoral anteversion and further guide the implantation of the femoral stem. All patients had an evident improvement in clinical scores and no severe complications occurred. Though our goniometer is not as accurate as computer navigation, it is easily put to practical use and less invasive. Above all, knowledge of the operative femoral anteversion can remind us of the need to adjust stem anteversion, further lowering the rate of prosthetic impingement.

Our study has several limitations. First, we did not validate the accuracy of our goniometer with a postoperative CT scan, which was not routinely examined after THA. Moreover, the discrepancy between intraoperative estimation and measurement on CT did not actually represent the precision due to the potential rotational adjustment of the femoral stem [25, 26]. Second, we did not concern about the actual posterior femoral condylar axis, but used the lower leg to approximate it. With the assumption that the lower leg is vertical to the posterior condylar axis, the orientation of its surface was not taken into consideration [15]. However, patients with advanced knee osteoarthritis were not included, which was the influencing factor for erroneous estimation. Third, no specific tools were available to measure the operative anteversion of the acetabular cup. We believe that it would not have much influence because of the error tolerance of our target combined anteversion [27, 28]. Fourth, although patients with various diagnoses were enrolled, the sample size of each might not be big enough.

The angle we called operative femoral anteversion is actually the torsional version on the cutting surface. We measured operative femoral anteversion not for femur first technique, but aimed to identify the abnormal native anteversion. We did not recommend adjusting the cup anteversion in tune with varying femoral anteversions. In cases with large native femoral anteversion, decreasing the cup anteversion can cause anterior protrusion of the cup due to the achievement of optimally combined anteversion. And impingement between the iliopsoas tendon and the anterior edge of the cup is a potential cause of groin pain and functional limitations after THA [29]. On the femoral side, we can adjust the stem anteversion to a physiologically normal value according to operative femoral anteversion. So we thought the operative anteversion was more meaningful for the planning of THA. To predict the position of the femoral stem, Park et al. built the relationships between native femoral anteversion on different CT sections and postoperative stem anteversion [13]. However, the version on the cutting surface was more visualized than the measurement results from preoperative CT scans. Influenced by the various lateral inclination of the femur, we can hardly make the measurement on a consistent CT section. Besides, the midcortical line of the cutting surface varied with the cutting height, which also increased the difficulty for preoperative estimation on CT scan [14, 30]. Therefore, we supposed that intraoperative estimation of femoral anteversion cannot be totally replaced by preoperative measurement. Several studies have found the lesser trochanter a reliable bony landmark. Based on a CT scan, Unlu et al. found a constant relationship between the version of less trochanter and posterior femoral condyles [31]. Shon et al. also observed a stable intersection angle between the posterior lesser trochanter line and femoral neck axis [32]. However, Worlicek et al. found significant differences in gender and left/right side when evaluating the correlation between the posterior lesser trochanter line and the posterior femoral condyle axis [33]. They concluded that posterior lesser trochanter line should not be used to determine femoral anteversion in CT scan. Moreover, different from measurement in the CT section, the contour of the lesser trochanter was difficult to determine due to its irregular morphology during THA, which further reduced its utility.

The operative femoral anteversion we measured ranged from −9 to 60°, with a large variation. There were not many results from previous literatures to compare. Researchers paid more attention to the estimation of stem anteversion, not the version of cutting surface [7, 15, 16, 18–20, 34]. Based on their results of stem anteversion, we can also find a wide range of values, which deviate from the generally advised 10–20° [16, 18, 19, 34]. It reminded us that variations of femoral proximal anatomy could be encountered in patients requiring THA. Different from other studies, we further analyzed the characteristics of hips with different ranges of femoral anteversion. Significant differences were observed in the age and diagnosis between hips with femoral anteversion >30° and the other two groups. Femoral anteversion >30° was more common in patients with a younger age and the diagnosis of DDH. This can be explained by the clinical features of DDH, including younger age when receiving THA and generally excessive femoral anteversion, especially for hips of low to high dislocation [35]. With regard to osteonecrosis, femoral anteversion <10° was seen in 15/49 hips, for whom care should be taken to prevent from inserting a relative retroverted stem. Generally, the anteversion of cementless femoral stem was thought to be dictated by the native proximal femoral anatomy, with less ability to adjust. But the tapered wedge stems we used in our cohort were demonstrated more flexible in rotation compared with metaphyseal fit stem [25, 26]. When there was a need of >10° adjustment, we preferred to choose the neck-adjustable stems. In this study, there were totally 14 hips treated with the neck-adjustable femoral stem during THA. Among them, there were 7 sleeve modular components and 7 monoblock stem with three different neck versions. We further analyzed the influencing factors for the need of neck-adjustable femoral stem. Based on the results of univariate analysis, we found that female sex, diagnosis of DDH (compared with osteonecrosis), and higher operative femoral anteversion and its value >30° (compared with <10°) were correlated with higher rates of using the neck-adjustable femoral stem. In sum, the primary influencing factor was the excessively larger femoral anteversion, which was more common in patients of DDH [35]. The discrepancy in sex can also be explained by the higher frequency of DDH pathology in women [36]. However, all factors lost statistical significance in the multivariate model. We supposed that it might be associated with the small sample size in the group of using neck-adjustable stem, which made the multivariate model unpowered to identify the important influencing factors. Moreover, apart from the concern about larger femoral anteversion, we should also actively deal with the anteversion <10° or even retroversion to avoid anterior impingement.

## Conclusions

This study introduced a newly developed goniometer to measure femoral anteversion intraoperatively and highlighted the significance of operative femoral anteversion. Identification of abnormal femoral anteversion could assist in adjusting stem anteversion and reduce the risk of dislocation after THA.

## Data Availability

All data generated or analyzed during this study are included in this published article.

## Abbreviations

THA: Total hip arthroplasty
DDH: Developmental dysplasia of the hip
BMI: Body mass index
OR: Odds ratio
CI: Confidence interval
